# A customizable method to characterize *Arabidopsis thaliana* transpiration under drought conditions

**DOI:** 10.1186/s13007-019-0474-0

**Published:** 2019-08-02

**Authors:** Carlos de Ollas, Clara Segarra-Medina, Miguel González-Guzmán, Jaime Puertolas, Aurelio Gómez-Cadenas

**Affiliations:** 1 Departamento de Ciencias Agrarias y del Medio Natural, Universitat Jaume I, Castellón de la Plana, Spain; 2 Lancaster Environment Centre, Lancaster University, Lancaster, UK

**Keywords:** ABA, Arabidopsis, Drought, Phenotyping, Transpiration

## Abstract

**Background:**

Characterization of the dynamic response of plant transpiration to decreasing soil water content in a reproducible way is required for the correct phenotyping of traits related to water saving strategies. Nowadays, an increasing number of automated high throughput platforms are available, but their development requires a great economic investment and it is not always desirable/feasible to outsource these analyses. We propose a medium-throughput protocol to characterize transpiration responses to decreasing soil moisture in a quantitative and highly reproducible way with a minimum investment of resources.

**Results:**

The quantitative characterization of plant responses to a decreasing soil water content using our phenotyping platform has showed high reproducibility between different experiments. The proposed irrigation strategy allowed us to harvest plants ranging from a well-watered condition to the loss-of-turgor point in a predictable and controlled way. Coupling this protocol with hormone profiling allows investigation of hormonal responses (metabolite accumulation as well as plant sensitivity) to water stress. As a proof-of-concept, we have characterized the dynamic responses of leaf transpiration to decreasing soil water contents in an abscisic acid (ABA) deficient genotype (*aba1*-*1*) as well as in genotypes with altered sensitivity to ABA (*abi1*-*1* and *hab1*-*1abi1*-*1*), which are insensitive and hypersensitive to ABA, respectively.

**Conclusions:**

This protocol allows for assessment of quantitative differences in rosette transpiration responses to water depletion in both ABA biosynthesis mutants and genotypes with altered sensitivity to the hormone. Data indicate a correlation between ABA levels and/or hormone perception and growth rate and/or water content. The protocol guarantees the correct application of water stress to adult plants, which is essential to understand responses of mutants and/or natural accessions.

**Electronic supplementary material:**

The online version of this article (10.1186/s13007-019-0474-0) contains supplementary material, which is available to authorized users.

## Background

Plant transpiration can be defined as the transport of water from the soil surrounding the roots to the aerial part of the plant and the subsequent evaporation from leaves or other organs. Most of plant water transpiration occurs through the leaf stomatal pores. Stomata aperture and, hence, transpiration is highly regulated by a plethora of both environmental (vapor pressure deficit (VPD), light, soil water content…) and metabolic (hormones, peptides, Ca^2+^…) signals [1–3]. The output of these signals regulates plant transpiration that in turn affect photosynthesis (as CO_2_ uptake depends of stomatal opening) and nutrition (as root water uptake, along with most inorganic salts, depends largely on the potential gradient between the atmosphere and the soil). These two key processes will roughly drive the overall plant metabolism and its performance. The balance of water spending and plant growth is defined by the water use efficiency or transpiration efficiency (TE, [4]). Due to the new environmental conditions caused by the climate change and the consequent worldwide food security alert, the search for traits to increase TE is necessary to obtain the maximum growth and/or yield with lower water consumption under both optimal and adverse growing conditions [5].

Despite the increasing number of articles reporting signal mediators for the stomatal closure [6], the hormone ABA is recognized as the key player controlling this process. On one hand, mutants defective in ABA biosynthesis such as *Solanum Lycopersicum flacca* [7] or *Arabidopsis thaliana aba1* [8] have a wilted phenotype with elevated stomatal conductance under both well-watered and drought conditions. On the other hand, mutants in ABA signalling show different sensitivities to the hormone leading to diminished or increased stomatal closure in response to variations in water availability/VPD changes. These variations depend on the signalling element and the nature of the mutation [9]. In WT plants, ABA concentration is tightly correlated to water status and soil moisture. Given this correlation, ABA biosynthesis and sensitivity are key targets to control transpiration and therefore water use efficiency.

We can measure transpiration at several scales (single leaf, whole plant, crop…) using a range of different techniques. For instance, leaf porometers only measure stomatal conductance (g_s_), while infra-red gas analysers can provide additional useful information on photosynthetic parameters. Nevertheless, the correct use of this equipment is not straightforward. Calibration and time of analysis per leaf can be a limiting factor when dealing with dozens or hundreds of samples. We must also consider that g_s_ is a dynamic response directly influenced by photoperiod, and so even with a good randomized design and enough replication, accuracy of the equipment can be overshadowed by circadian variations amongst other factors. Another problem arises when measurements taken from different types of leaves are assumed to be representative of whole plant transpiration, resulting in further variation. This forces us instead to make measurements in similar leaves throughout plants. However, if we are not primarily interested in photosynthesis, whole plant gravimetric transpiration can be a more practical way of measuring transpiration if we isolate soil evaporation from plant transpiration and normalize the projected leaf area. Hence, there is the need to measure leaf area in a non-destructive way, which is nowadays easy due to the widespread use of high-quality cameras in smartphones and free image analyser software as Easy Leaf Area [10].

Phenotyping plant responses to water stress is not a straightforward task for several reasons beginning with the definition of stress and the quantitative measurements necessary to characterize the environmental conditions and finishing with the interpretation of results. Water stress experiments should distinguish between plants presenting dehydration avoidance or tolerance strategies [11]. The use of environmental chambers offers a controlled environment in terms of light and relative humidity but not regarding substrate water content and distribution, which are key elements to account for water availability. In this sense, a homogeneous substrate in terms of composition and water release properties is an important requirement. Soil moisture/relative water content must be monitored throughout the experiment to pair these data with plant physiological/analytical measurements [12]. Another critical point is the irrigation strategy to reach a given low soil moisture goal in a reproducible way, which is commonly performed by measuring the soil water content and replenishing the water loss to the given predetermined low content. In terms of physiological responses, this strategy can cause short daily cycles of hydration/dehydration with unreal and unpredictable consequences [13, 14]. Plant morphological characterization (size, leaf area, number of leaves, growth rate…) is also necessary in a dynamic (hence non-destructive) way, as soil water uptake rate is primary influenced by plant size/leaf area. When comparing mutants or ecotypes with different sizes, it must be considered that smaller plants uptake and transpire water from the substrate at a slower rate. Moreover, water stress is not a single condition but a process with multiple stages ranging from a well-watered plant to a plant that has completely lost turgor (and eventually will die) due to a water uptake insufficient to match transpirational water loss. To identify plant stress responses, we need to characterize this dynamic process influenced by the severity of the stress condition (water content, temperature and VPD) and time of exposure to the condition.

Automated phenotyping platforms such as Phenopsis [15] allow for obtaining this information for thousands of plants per batch with a reasonably small technical intervention, but at huge economic cost. Although it is possible to outsource characterization of lines of interest to these platforms, *in*-*house* characterization would reduce the cost of analysis. Thus, we have designed a simple non-automated medium-throughput (order of hundred plants per batch) protocol with reduced economical investment to characterize plant transpiration phenotypes. We have coupled physiological and morphological results with hormone profiling analysis to characterize dynamic responses in ABA deficient (*aba1*-*1*), ABA insensitive (*abi1*-*1*), and ABA hypersensitive (*hab1*-*1 abi1*-*2*) lines as proof of concept, highlighting protocol strengths and weaknesses.

## Results

### Gravimetric characterization of whole plant transpiration under decreasing soil water content

In these experiments we have characterized whole plant daily transpiration under decreasing soil water content ranging from 0.4–0.5 to 0.1 g g^−1^ of soil water content (SVWC, g of water/g of soil), which is consistent with short term wilting after progressive desiccation. To have a practical and reproducible soil water content we used individual peat plugs, that once isolated with a shell (pots) represent a small, easy-to-carry portion of substrate that has low variation (within and between batches) in physical properties. Low variation in those physical properties allows to transform SVWC to soil water potential of individual pots using the equation calculated with the water release curve data (Additional file 1: Figure S1).

**Fig. 1:**
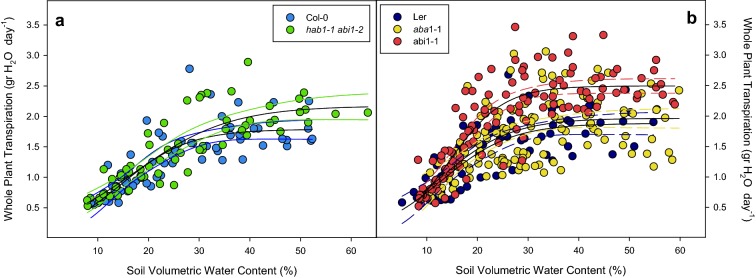
Whole plant daily transpiration versus soil volumetric water content (SVWC) of **a** Col-0 (blue dots), *hab1*-*1 abi1*-*2* (green dots) and **b** L*e*r (dark blue dots), *aba1*-*1* (yellow dots) and *abi1*-*1* (red dots). Points represent individual transpiration values across four different days prior to harvesting. Blank solid lines represent fitting to sigmoid functions. Coloured lines represent 95% confidence bands of each fitting line. Significance of these results (ANOVA) is summarized in Table 1

Raw daily transpiration per plant ranged from 1.5 to 2.0 ml of water/day in WT accessions (Col-0 and L*er*, Fig. 1) under well-watered conditions (pot weight above 25 g; SVWC > 0.4) and significantly decreased due to lower SVWC dropping to 0.5 ml/day and leading to loss of leaf turgor and plant wilted phenotype. The double mutant *hab1*-*1 abi1*-*2* (in Columbia-0 background (Col-0), Fig. 1a) and mutants *aba1*-*1* and *abi1*-*1* (in Landsberg *erecta* background (L*er*), Fig. 1b) showed a similar range of raw transpiration values, and only *abi1*-*1* had higher transpiration than L*er* despite its average shorter size. On the other hand, *aba1*-*1* mutant showed a huge scattering of transpiration values throughout SVWC range. Transpiration values for all lines converged to a sigmoid function without further rosette area normalization.

### Plant transpiration per unit of area under decreasing soil water content

**Fig. 2:**
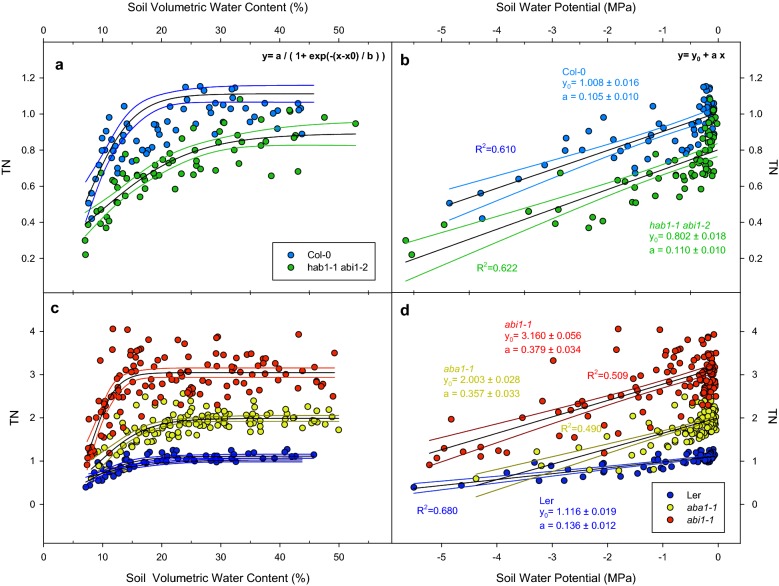
Daily transpiration normalized to leaf area and Col-0 maximum transpiration (TN) versus soil volumetric water content (SVWC; **a**, **c**) or soil water potential (SWP [MPa]; **b**, **d**) of Col-0 (blue dots), *hab1*-*1 abi1*-*2* (green dots), L*e*r (dark blue dots), *aba1*-*1* (yellow dots) and *abi1*-*1* (red dots). Points represent individual TN values across four different days prior to harvesting. Blank solid lines represent fitting to sigmoid (**a**, **c**) or linear (**b**, **d**) functions. Coloured lines represent 95% confidence bands of each fitting line. Significance of these results (ANOVA) is summarized in Table 1

To consider the influence of rosette area size (both within and between genotypes) on plant transpiration, individual rosette projected area was used to normalize whole plant transpiration per unit of area (mm^2^). Normalized transpiration (TN) was recorded over 4 (hence three replicates) consecutive days to obtain repeated measurements of the same pots as water was transpired by the plants (Fig. 2). To pool and plot together results of these days, all values of the same day were normalized according maximum transpiration of Col-0 plants under well-watered conditions (Fig. 2a, c) and therefore, TN is presented with no units. On one hand, Col-0 TN under well-watered conditions was 1.02 ± 0.02, whereas *hab1*-*1 abi1*-*2* TN was lower (0.89 ± 0.03) and under decreasing SVWC, the transpiration of both genotypes decreased at similar rate (Fig. 2a). On the other hand, L*er* had transpiration values slightly higher than Col-0 (1.11 ± 0.02) and both *aba1*-*1* and *abi1*-*1* showed high transpiration rates (1.98 ± 0.03 and 3.04 ± 0.05, respectively, Fig. 2c).

### Plant transpiration per unit of area under decreasing soil water potential

By plotting transpiration versus the soil water potential (SWP) we aimed to simplify the non-linear relationship (sigmoid) into a linear one. To achieve this, we characterized the relationship between SVWC and SWP, calculating the equation that describes their relationship (Additional file 1: Figure S1). As expected, transpiration values for all the Arabidopsis lines plotted against SWP fitted into linear functions in the studied range (Fig. 2b, d). Col-0 and *hab1*-*1 abi1*-*2* transpiration rates decreased in parallel [no significant differences in slopes after ANCOVA (Table 1)] from different starting (well-watered) points defined by the intercept of the linear function. Col-0 TN was higher than *hab1*-*1* *abi1*-*2* (1.00 ± 0.01 and 0.80 ± 0.02, respectively; Fig. 2b), *aba1*-*1* and *abi1*-*1* had significantly higher TN than L*er* (1.11 ± 0.02) under high SWP (2.00 ± 0.02 and 3.16 ± 0.05, respectively). Rates of TN change with SWP (defined by the slopes of lines in Fig. 2 D) were higher in *aba1*-*1* and *abi1*-*1* (0.35 ± 0.03 and 0.37 ± 0.03, respectively) than in L*er* (0.13 ± 0.01).

**Table 1:** Significance (p-values) after ANOVA of results depicted in Figs. 1, 2, 3, 4, 5, 6 (first column)

		Col-0 VS *hab1*-*1 abi1*-*2*	L*er* VS *aba1*-*1* VS *abi1*-*1*	Ler VS *aba1*-*1*	Ler VS *abi1*-*1*	*aba1*-*1* VS *abi1*-*1*
Figure 1	Whole plant transpiration					
Factor	Genotype	0.938	< 0.001			
Covariate	Soil volumetric water content	< 0.001	< 0.001			
ANCOVA	G × SWP	0.339	< 0.001	0.099	< 0.001	< 0.001
Figure 2	Transpiration normalized					
Factor	Genotype	< 0.001	< 0.001			
Covariate	Soil water potential	< 0.001	< 0.001			
ANCOVA	G × SWP	0.730	< 0.001	< 0.001	< 0.001	0.622
Figure 3	Leaf relative water content					
Factor	Genotype	0.007	< 0.001			
Covariate	Soil Water Potential	< 0.001	< 0.001			
ANCOVA	G × SWP	0.836	0.458	0.182	0.444	0.530
Figure 4	ABA					
Factor	Genotype	0.005	< 0.001			
Covariate	Soil water potential	< 0.001	< 0.001			
ANCOVA	G × SWP	0.062	< 0.001	< 0.001	< 0.001	< 0.001
Figure 5	Transpiration normalized					
Factor	Genotype	< 0.001	< 0.001			
Covariate	ABA	< 0.001	< 0.001			
ANCOVA	G × ABA	0.047	< 0.001	< 0.001	0.418	< 0.001
Figure 6	Shoot fresh weight					
Factor	Genotype	< 0.001	< 0.001			
Covariate	Soil water potential	< 0.001	< 0.001			
ANCOVA	G × SWP	0.372	0.622	0.746	0.341	0.132
Shoot dry weight						
Factor	Genotype	0.003	< 0.001			
Covariate	Soil water potential	< 0.001	0.033			
ANCOVA	G × SWP	0.203	0.324	0.374	0.136	0.908
Relative growth rate						
Factor	Genotype	0.593	0.009			
Covariate	Soil water potential	< 0.001	< 0 .001			
ANCOVA	G × SWP	0.260	0.001	0.003	0.005	0.159

Factors used to build the model (independent/dependent/covariate and interaction; second column)

### Leaf relative water content under decreasing soil volumetric water content/soil water potential

**Fig. 3:**
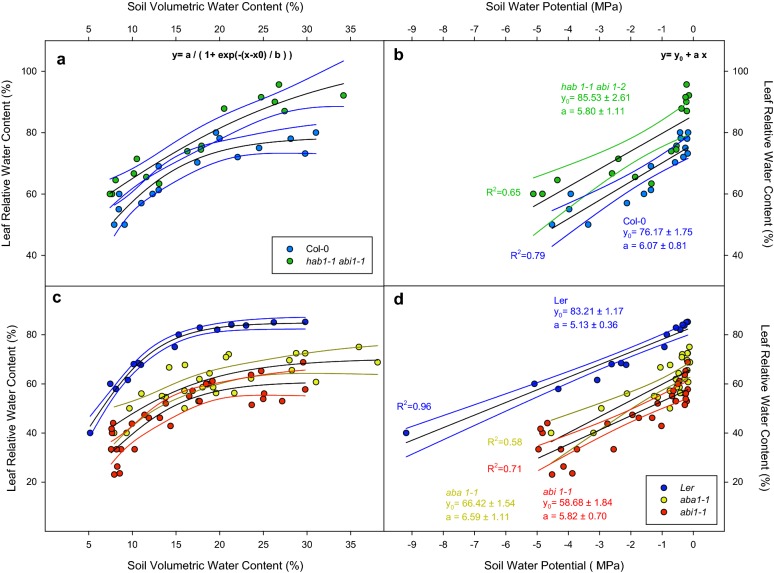
Leaf relative water content (LRWC) versus soil volumetric water content (SVWC; **a**, **c**) or soil water potential (SWP [MPa]; **b**, **d**) of Col-0 (blue dots), *hab1*-*1 abi1*-*2* (green dots), L*er* (dark blue dots), *aba1*-*1* (yellow dots) and *abi1*-*1* (red dots). Points represent individual LRWC values immediately prior to harvesting. Blank solid lines represent fitting to sigmoid (**a**, **c**) or linear (**b**, **d**) functions. Coloured lines represent 95% confidence bands of each fitting line. Significance of these results (ANOVA) is summarized in Table 1

Leaf relative water content (LRWC) in equivalent leaves of all plants was measured to account for the leaf water status in our experimental range of SVWC/SWP (Fig. 3). Unlike transpiration, LRWC measures are single destructive measurements during the harvesting and normalization of data was not necessary to compare water status dynamics. Data of LRWC versus SVWC fitted into sigmoid functions for Col-0 and *hab1*-*1 abi1*-*2* although these genotypes had different LRWC under high SVWC (Fig. 3a). However, under low SVWC differences were reduced. L*er* had similar LRWC than Col-0 in contrast to *aba1*-*1* (70.2 ± 3.6) and *abi1*-*1* (60.8 ± 3.0) that showed lower values (Fig. 3b). Under decreasing SVWC, LRWC declined in all lines converging to similar values. After linear fitting of LRWC versus SWP (Fig. 3b, d), the double mutant *hab1*-*1 abi1*-*2* had significantly higher LRWC (85.5 ± 2.6 g g^−1^) at high SWP compared to Col-0 (76.2 ± 1.7 g g^−1^) but with similar regression slopes (6.1 ± 0.8 in Col-0 versus 5.8 ± 1.1 g g^−1^ MPa^−1^ in *hab1*-*1 abi1*-*2*). L*er* had similar LRWC (83.2 ± 1.2 g g^−1^) than Col-0 and both *aba1*-*1* and *abi1*-*1* single mutants showed lower LRWC values with no differences between their slopes (Fig. 3d).

### ABA accumulation under decreasing soil volumetric water content and soil water potential

**Fig. 4:**
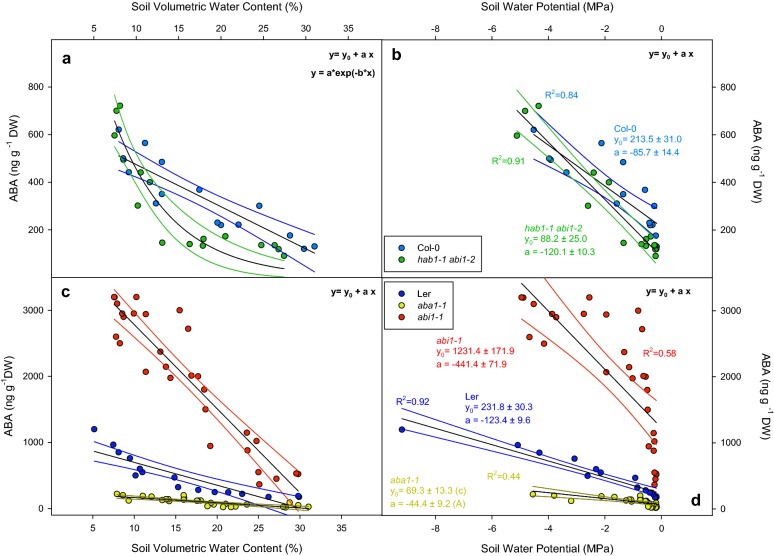
Leaf ABA concentration (ng g^−1^ DW) versus soil volumetric water content (SVWC; **a**, **c**) or soil water potential (SWP [MPa]; **b**, **d**) of Col-0 (blue dots), *hab1*-*1* *abi1*-*2* (green dots), L*er* (dark blue dots), *aba1*-*1* (yellow dots) and *abi1*-*1* (red dots). Points represent individual endogenous ABA concentrations at the moment of harvesting. Blank solid lines represent fitting to linear functions except in Col-0 in **a** that fitting follows an exponential decay function. Coloured lines represent 95% confidence bands of each fitting line. Significance of these results (ANOVA) is summarized in Table 1

ABA levels accumulated linearly in Col-0 under decreasing SVWC (from 130 to 620 ng g^−1^ DW, Fig. 4a) and they increased in a similar range in *hab1*-*1 abi1*-*2* but, unlike Col-0, ABA levels versus SVWC did not fit a linear function (Fig. 4a). Hence, we used an exponential decay function to suggest a possible difference in accumulation kinetics; however, this does not allow a parametric comparison. On the other hand, in L*er*, *aba1*-*1* and *abi1*-*1*, hormone concentration versus SVWC did fit a linear function (Fig. 4c). Similar to Col-0, L*er* plants had ABA concentrations ranging from 176 to 614 ng g^−1^ DW, *aba1*-*1* plants had lower ABA concentrations than L*er*, ranging from 25.03 to 231.15 ng g^−1^ DW. Levels in *abi1*-*1* were the highest both under well-watered conditions (485.10 ng g^−1^ DW) and under water scarcity (3235.91 ng g^−1^ DW) and the ABA accumulation rate was significantly higher in *abi1*-*1* than in any other genotype.

Like TN, endogenous ABA concentrations fitted linear functions once plotted versus SWP for each genotype (Fig. 4b, d). Initial ABA concentration (well-watered conditions) was lower in *hab1*-*1 abi1*-*1* (88.2 ± 25.0 ng g^−1^ DW) compared to Col-0 (213.5 ± 31.0 ng g^−1^ DW). In L*er* both ABA concentration and accumulation rate were significantly different from *aba1*-*1 and abi1-1* mutants (Table 1); L*er* had ABA concentrations of 231.8 ± 30.3 ng g^−1^ DW whereas *aba1*-*1* (69.3 ± 13.3 ng g^−1^ DW) and *abi1*-*1* (1231.4 ± 171.9 ng g^−1^ DW) had lower and higher ABA concentrations respectively. Compared to L*er*, slopes of linear functions were also lower and higher for *aba1*-*1* and *abi1*-*1*, respectively (P < 0.001) after ANCOVA (Table 1).

The main advantage of plotting ABA versus SWP instead of SVWC relates to the reduction of SVWC range comprising well-watered conditions that correspond to a narrow range of negative potentials close to zero (Fig. 4b, d). At high SWP values ABA concentrations are lower in *hab1*-*1 abi1*-*2* plants, but the slope of the accumulation line is higher in the case of *hab1*-*1 abi1*-*2* compared to Col-0 (− 120.2 vs. − 85.7, respectively).

### Endogenous ABA effect on plant transpiration

**Fig. 5:**
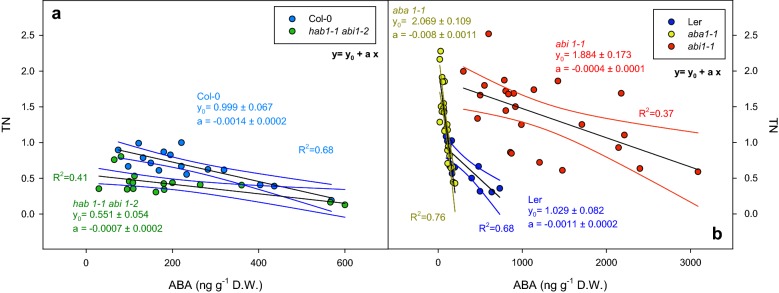
Transpiration normalized to leaf area and Col-0 maximum transpiration (TN) versus leaf ABA concentration (ng g^−1^) of **a** Col-0 (blue dots), *hab1*-*1 abi1*-*2* (green dots) and **b** L*er* (dark blue dots), *aba1*-*1* (yellow dots) and *abi1*-*1* (red dots). Points represent individual endogenous ABA concentrations paired with the ETN of each individual plant. Black solid lines represent fitting to linear functions. Coloured lines represent 95% confidence bands of each fitting line. Significance of these results (ANOVA) is summarized in Table 1

We plotted endogenous ABA versus TN to obtain quantitative information about genotypic variation in transpiration sensitivity related to the endogenous ABA concentration increase (the ratio of TN to endogenous ABA levels, that corresponds to the slope of the linear function). The slope of this relationship in the Col-0 genotype (Fig. 5a) was significantly higher than in *hab1*-*1 abi1*-*2* (P = 0.047). However, Fig. 5b shows a significantly different relationship of the endogenous ABA concentration over transpiration (P < 0.001) among Ler, *aba1*-*1* and *abi1*-*1*, with *abi1*-*1* showing the lowest slope values (matching with the insensitivity of this genotype to ABA). Interestingly, the different slope of *aba1*-*1* compared to L*er* at low ABA concentrations might reflect an altered sensitivity to ABA in that low concentration range.

### Influence of drought stress on plant growth parameters

**Fig. 6:**
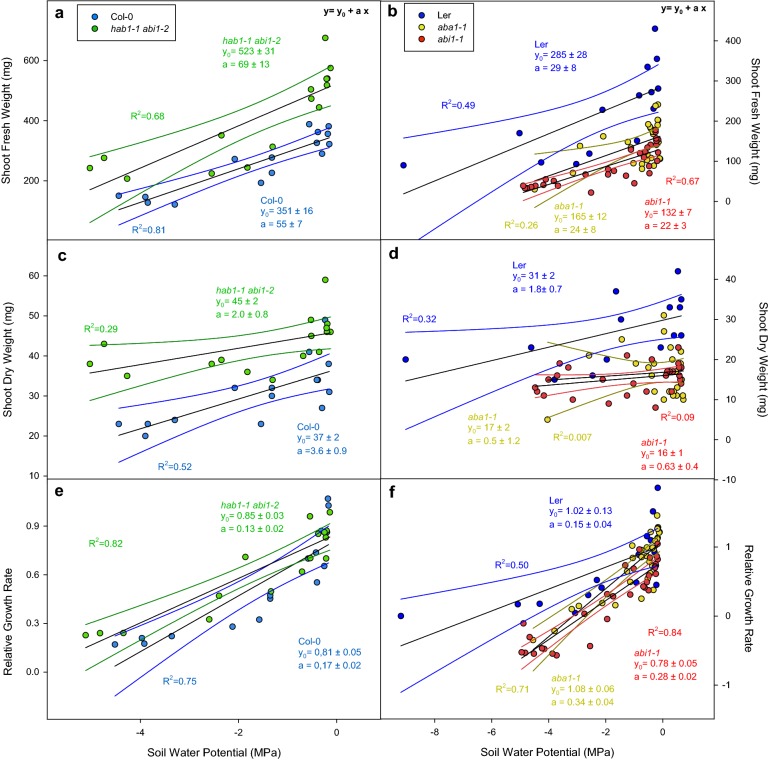
Shoot fresh weight (SFW **a**, **b**), shoot dry weight (SDW **c**, **d**) and relative growth rate (RGR **e**, **f**) versus soil water potential (SWP, MPa) of Col-0 (blue dots), *hab1*-*1 abi1*-*2* (green dots), L*er* (dark blue dots), *aba1*-*1* (yellow dots) and *abi1*-*1* (red dots). Points represent individual values paired with the SWP of each individual plant. Black solid lines represent fitting to linear functions. Coloured lines represent 95% confidence bands of each fitting line. Significance of these results (ANOVA) is summarized in Table 1

To study the effect of genetic and environmental factors over plant growth, as well as the interaction between genotype and drought stress, we recorded rosette fresh and dry weight (SFW and SDW respectively) and relative growth rate (RGR) by means of projected leaf area measurements. Figure 6 shows the values of SFW (ab), SDW (cd) and RGR (ef). Plant fresh weight is a result of the combination of tissue growth history and its current water content. Measurements of dry weight is only dependent on overall growth since germination. Growth rate, on the contrary only reflects rosette expansion by relative variation in projected leaf area throughout the stress treatment period monitored.

Col-0 had lower SFW than *hab1*-*1 abi1*-*2* at high SWP (Fig. 6a). Under decreasing SWP the SFW of both genotypes decreased linearly with a similar rate (Table 1, p-value = 0.372). On the contrary both *aba1*-*1* and *abi1*-*1* had lower SFW than L*er* (Fig. 8b), with a similar SFW decrease rate in all three genotypes (p-value = 0.622).

Similarly, Col-0 had lower SDW than *hab1*-*1* *abi1*-*2* at high SWP (Fig. 6c) and SDW decreased in both genotypes with a similar rate under drought stress conditions. L*er* had higher SDW than *aba1*-*1* and *abi1*-*1* but SDW decreased at a similar rate under lower SWP (p-value = 0.324) in all genotypes.

Relative growth rate was recorded from the day of the last watering before the experiment to the harvesting day (after 6 days). Col-0 had similar RGR than *hab1*-*1* *abi1*-*2*, decreasing with a similar rate both genotypes under stress conditions (Fig. 6e). However, L*er* and *aba1*-*1* had higher RGR than *abi1*-*1*. Interestingly, under stress conditions all these genotypes suffered a decrease in their RGR but with different magnitude, with a minor decrease in growth rate in L*er* compared to *aba1*-*1* and *abi1*-*1* (Fig. 6f).

## Discussion

Phenotyping plant responses to water deficit is a complex task due to the existence of multiple interactions between morphology (size and anatomical differences in root and shoot architecture…), natural variation in biochemical responses and signalling networks that operate in complex feedbacks. The dynamic interaction of the plant with the atmosphere and the soil substrate that holds the water is also difficult to standardize and quantify; therefore, is common to mislabel genotypes as tolerant, sensitive or resistant when these parameters are not precisely standardized and quantified. Although there is a good consensus on the key processes and players (molecular, hormonal, morphological…) involved in plant responses to water shortage, the quantitative characterization of these phenotypes is deficient or even absent quite often. The aim of this work is to offer a simple but highly reproducible workflow to characterize early responses to water stress in Arabidopsis mutants and ecotypes.

One essential aspect for establishing this platform was controlling water content of the substrate in a reproducible but practical way. Individual peat plugs allowed to achieve this requirement as: (a) they avoid interaction between plant water withdrawal of a shared substrate and (b) have a small, easy-to-carry portion of substrate that has low variation (within and between batches) in physical properties such as weight and field capacity (Additional file 1: Figure S2). Once characterized, the water release curve from the peat plugs (Additional file 1: Figure S1), showed a good correlation (R^2^ = 0.9700) between plug weight and volumetric water content or soil water potential.

The use of an infrared gas exchange analyser is one of the most popular and accurate ways of measuring instant transpiration. Although it has multiple advantages, it is also time consuming and accuracy of the value gets compromised when analysis is extended more than a few hours due to circadian changes in plant stomatal conductance. A practical alternative is to calculate water loss in a time interval by weighing pots but for this method to be accurate the soil must be as isolated as possible from the environment to avoid direct water loss from the soil to the atmosphere (that can be in the same order of magnitude than water transpired by the plant) yet allowing the plant to grow undisturbed. In these conditions, differences in weight mostly account for water transpired by the plant. To achieve this situation, plugs were isolated using adapted plastic cups in which small plants (second true leaf stage) can remain undisturbed throughout the isolation process. This was an effective and low-cost strategy to cover the plug although more sophisticated shells probably can be obtained by using 3D printing technology. Once the plug was isolated with duct tape, soil evaporation was almost abolished (95% reduction compared to plugs without shell, Additional file 1: Figure S3), making feasible to measure whole plant transpiration in a reproducible way.

A critical aspect of this workflow is to find the optimal plant size/age to characterize plant response to decreasing soil water content. If plants are too small (from second leaf to 6–8 leaves, 2 weeks after germination) the signal (whole plant transpiration) to noise (soil evaporation) ratio will be suboptimal. On the contrary, if plants are too old and large, whole plant transpiration will be large as well. This implies lower resolution in the x-axis once transpiration is plotted versus SVWC or SWP. In an extreme case, a fully developed plant (from 5 weeks to flowering under short day) can transpire 4–5 ml a day in our environmental conditions. This means that the plant can go from a fully-hydrated status (pot weight around 20–25 g and SWP near cero) to a wilting point (pot weight of 10–13 g and SWP = − 3 MPa) in 2 days. If the transpiration is measured daily then we only have two steps from a well-watered to a wilted plant. The optimal point between these two extremes (low plant transpiration gives high resolution in the x-axis but soil transpiration causes noise in the y-axis and on the contrary high transpiration gives low x-axis resolution and low noise in the y-axis) was found at 22–27 days after germination (stage 1.10 [16]) when plants of all tested genotypes had daily transpirations of 1.5–2 ml (Fig. 1).

Whole plant transpiration (an extensive property) must be normalized with each plant rosette area to distinguish differences in an intensive property (as TN) due to genotype and treatment. Nowadays, there are plenty options to quantify leaf area in a non-destructive way by recording projected leaf area [17]. In this work two free available software (Easy Leaf area and Leaf-GP [10, 19]) were used with a smartphone camera with common laboratory illumination and a plain white background. Both software are good options although in our conditions Easy Leaf Area had a better performance in the rosette segmentation process, the output file was then used to further analysis with Leaf-GP which offers the quantification of an interesting number of morphological parameters.

Once leaf transpiration was normalized to the rosette area (TN) for each plant, the method had enough precision to distinguish differences in TN between Col-0 and *hab1*-*1* *abi1*-*2* (which is about a 20% lower throughout the dehydration period) or L*er* (10% increase compared to Col-0). An important advantage of working with SWP as a continuous (co-variant) factor is that the slope of the SWP vs transpiration regression line can be also calculated. This slope defines the change of transpiration per unit of soil water potential, which we can be used as a proxy to screen genotype sensitivity to soil desiccation in a quantitative way. For instance, the slopes of TN vs SVWC relationships for *aba1*-*1* and *abi1*-*1* were significantly higher than that of L*er*, indicating a higher sensitivity to soil dryness.

Transpiration, LRWC and ABA quantification results (Figs. 1, 2, 3) have been plotted versus SVWC and SWP to show the raw data obtained from weighing the pots and the transformed data into a linear relation with SWP. Data were fitted to a linear function because the statistical treatment is easier compared to nonlinear functions but also because water potential is a more comparable (between different types of substrate) parameter than SVWC [18].

Plant water status relies on the equilibrium between water uptake from the soil and water loss to the atmosphere (mostly through stomata) [19]. LRWC is an indicator of a genotype ability to maintain a high-water status. In this sense, *hab1*-*1* *abi1*-*2* had higher LRWC than Col-0 in the studied range of SWP (Fig. 3b), presumably through a more closed stomata that allows to avoid water loss [20]. On the other hand, both *aba1*-*1* and *abi1*-*1* had low LRWC values even under SWP close to zero (Fig. 3d). Timing and precision for leaf sampling to calculate LRWC is crucial. Plant material must be harvested in a narrow time window (3 h at the most) starting at a fixed time after the beginning of the photoperiod since water content fluctuates during the day, achieving maximum values at night (due to stomata closure). Once lights open the stomata, plants under suboptimal water availability suffer a decrease in LRWC as the day elapses [21]. A randomized block design (genotypes × treatments) is necessary for an unbiased harvesting strategy to overcome circadian changes and get consistent data. Another important detail is leaf selection, equivalent leaves must be harvested (mature fully expanded leaves in this work) between plants to calculate LRWC as age/position of the leaf will affect its LRWC [22].

One of the main players in plant responses to water shortage is the hormone ABA. Mutants deficient in ABA biosynthesis or signalling offer an illustrative array of phenotypes that surprisingly have not been yet completely characterized [23]. Both ABA-deficient (*aba1*-*1*) and ABA-insensitive (*abi1*-*1*) mutants have constitutive stomatal opening, a wilted phenotype and impaired growth [24]. Figure 4a, d shows linear ABA accumulation under decreasing SWP in all studied genotypes. Interestingly, *hab1*-*1* *abi1*-*2* had lower ABA levels compared to Col-0 under well-watered conditions but its rate of accumulation with soil drying (slope of the ABA vs SWP linear regression) was higher (similarly to *abi1*-*2*). This reflects how this method allows screening for quantitative differences in the ratio of hormone accumulation and sensitivity to reproducible values of SVWC/SWP.

On top of biosynthesis, sensitivity to endogenous ABA concentration is an important factor when phenotyping stress responses. This important parameters usually gets overlooked even when endogenous hormone concentration is quantified [25]. Therefore, in this work it is shown that initial transpiration values are different between Col-0 and *hab1*-*1* *abi1*-*2* although ABA concentration is similar (Fig. 5a). Moreover, ABA levels necessary to reduce transpiration in these genotypes differ (as it can be observed for the different slopes of the lines after ANCOVA, Table 1). For instance, *hab1*-*1* *abi1*-*2* needs lower ABA levels to reduce transpiration than Col-0 (Fig. 5a) but *abi1*-*1* needs to accumulate up to 3000 ng g^−1^ of ABA to reduce transpiration to levels displayed by L*er* under control conditions. These results show the importance of using SVWC as a continuous factor and the usefulness of paired measurements of physiological responses and hormonal quantification for each individual plant.

Genotype growth rate, environmental conditions and its interaction are important parameters to select and characterize tolerance to stress. Genotypes able to withstand growth under stress pressure can be models for obtaining more efficient crops. In this sense, measurements of shoot fresh and dry weight and relative growth rate seem a good starting point for selection of these traits. Shoot fresh weight is a result of the plant growth since germination and its current water content. However, shoot dry weight is entirely related to plant growth since germination. Finally, relative growth rate indicates differences in leaf expansion of plants under different SVWC conditions. In this work *hab1*-*1* *abi1*-*2* had higher weight (both fresh and dry) than Col-0 under well-watered conditions; however, the rate of decrease in RGR with SVWC was similar to Col-0 (Fig. 6a, c) and showed the same trend than the RGR (Fig. 6e) in both genotypes. These results indicate that *hab1*-*1* *abi1*-*2* has a greater growth potential under well-watered conditions. However a prolonged stress treatment would be necessary to observe robust differences in RGR and confirm the better performance of this genotype under stress. On the other side, both *aba1*-*1* and *abi1*-*1* had lower weight (fresh and dry) but these parameters decreased with an increasing SVWC at a similar rate than L*er*. Although RGR was different between L*er* and *aba1*-*1*/*abi1*-*1*, the negative values in growth rate at low SWP point to one of the limitations of this method, as these genotypes wilt under relatively high SWP (Fig. 3d). Therefore, leaf area under these circumstances does not reflect the real growth that probably should be assumed as zero.

## Conclusion

We propose this pipeline to screen Arabidopsis genotypes (mutants and natural accessions) with differences in transpiration under well-watered and under well-defined water limiting conditions. On top of transpiration, recording growth parameters allow to screen for genotypes with differential tolerance to stress or avoidance strategies. The great advantage of an easy an inexpensive protocol is that it can be custom upgraded with particular laboratory techniques to have a proper framework to study water stress. For example, this method can be useful for multihormone monitoring and metabolomic transcriptomics platforms. Imaging techniques and hyperspectral technologies are also complementary phenotyping strategies to obtain quality data and build more accurate models.

## Materials and methods

### Plant material and growth conditions

**Fig. 7:**
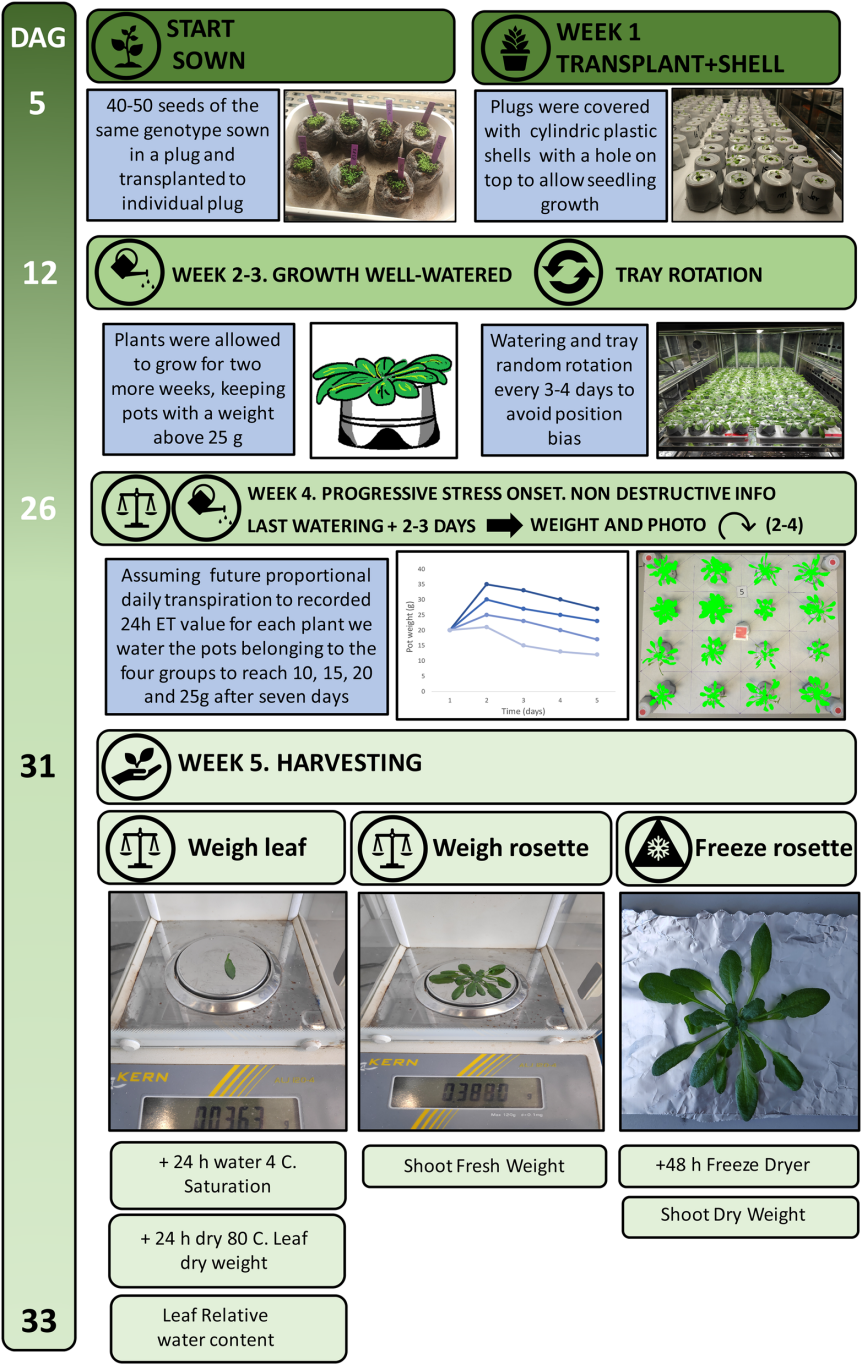
Workflow of the protocol depicting the most important steps from germination to harvesting

The double *hab1*-*1 abi1*-*2* mutant and its genetic background Columbia-0 were originally described in [26]; the single *aba1*-*1* and *abi1*-*2* mutants in L*er* genetic background were described by [27] and have been previously characterized in our laboratory [24]. Seeds (50–100) of each individual line were sown in peat plugs (Jiffy-7 peat pellets, Semillas Batlle S.A., Barcelona, Spain) without further stratification. Five days after germination, individual seedlings were carefully transplanted to plugs with tweezers, and were kept for a week in a growth chamber (Equitec model EGCS 351 3S HR, Getafe, Spain) with a day/night temperature of 23/18 °C, a 8 h light photoperiod (100 μmol m^−2^ s^−1^), and a relative humidity of 60–65%. After 5–7 days growing into the growth chamber, 16 (Col-0, Ler, *hab1*-*1* *abi1*-*2*) or 32 (*aba1*-*1* and *abi1*-*1*) plants showing homogeneous growth were selected for the experiment. Plugs were covered with a bottomless cylindric plastic shell to avoid soil evaporation (measures and photographs are provided in Additional file 1: Figure S4) with a hole on top to place the seedling (pot). The bottom of these cylindric plastic shell was sealed with a 4 × 4 cm piece of duct tape exposed to a gentle flame. Pots were randomly distributed in 20 × 20 × 1 cm polystyrene trays (16 plants per tray). Pot position within a tray was fixed (1–16) and each tray was labelled (1–7). Plants were cultivated for 2 more weeks, rotating the trays within the growth chamber every few days to avoid any position bias. A schematic of the process is summarized in Fig. 7.

### Stress treatment and harvesting

To calculate initial plant daily transpiration, once plant growth was optimal, pots were weighed between 9 and 11 h am and once again after 24 h. Plants were assigned to four different groups (four or eight plants per group) and, assuming a proportional daily transpiration, each pot was watered that day to reach different weights (10, 15, 20 and 25 g of water equivalent to a soil volumetric water content of 10, 20, 40 and 50%) after 6 days, according to this formula [Water (ml) = Goal pot weight (g) + (daily transpiration (g) × 7 (days)) − current pot weight (g)]. We took photographs of each plant during the 4 days that weights were scored (Additional file 2) to calculate projected leaf area and other morphological parameters using pixel count references according to the Easy Leaf Area [10] and Leaf-GP software [17]. Weight and projected leaf area values were used to calculate daily transpiration for each plant. Results of consecutive days were normalized according to the mean transpiration of the four Col-0 plants (considered as value one) on well-watered control conditions (25 g pot weight). The other parameters obtained from Leaf-GP as leaf perimeter or number of leaves can be found in Additional file 3.

**Fig. 8:**
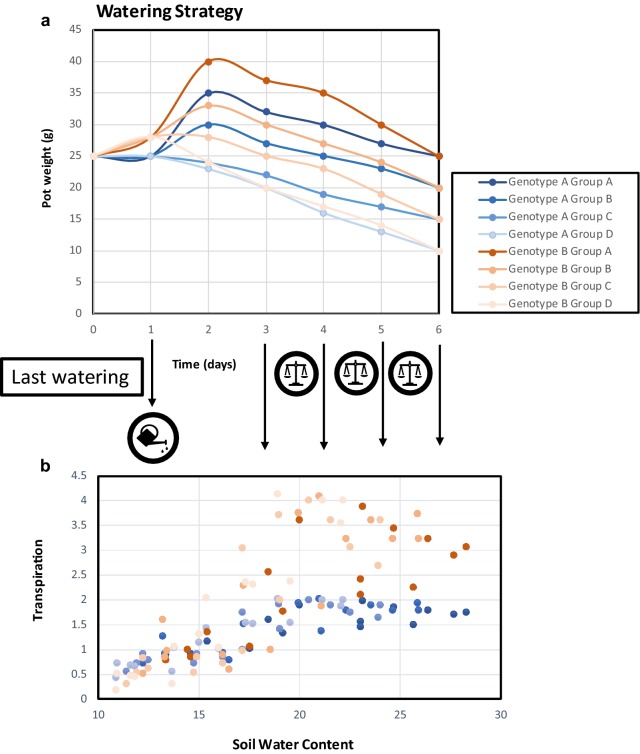
Theoretical representation of stress irrigation strategy. **a** Plot of pot weight (substrate + water) in two genotypes (A-blue, B-red) watered in arbitrary day 1 to reach four different water content goals (25, 20, 15 and 10) after 6 days, divided in Group A (well-watered) to Group D most severe stress. **b** Sequential measurements of daily transpiration of different genotypes corresponding to measurements throughout stress treatment to harvesting (arbitrary day 6)

After the final round of weighing, plants were harvested (Fig. 8 summarizes the watering strategy and harvesting). A fully developed leaf of each plant was excised with tweezers, weighed (fresh) with an analytical balance (ALJ120-4, Kern, Balingen, Germany) and placed in a 2 ml eppendorf tube filled with tap water which was kept at 4 °C in the darkness for 24 h. After that period, leaves were weighed again to score the leaf saturation weight. Then, leaves were placed into an oven at 60 °C for another 24 h and weighed afterwards to calculate the relative water content (RWC) of each leaf according to the formula RWC (%) = ((Fresh weight − dry weight)/(Saturated weight-dry weight)) × 100. Additionally, the whole rosette was excised and weighed to score the rosette fresh weight, gently wrapped in aluminium foil, labelled and placed in liquid N_2_. This frozen rosette material was lyophilized (Telstar Lyoalfa L-6-80, Telstar, Terrassa, Spain) and weighed afterwards to calculate dry weight. Additional files 4 and 5 contain raw data of an independent experiment covering all results shown throughout the paper.

### Hormone analysis

Ten mg of dry material was used for hormone analyses performed according to [28] with slight modifications. Briefly, 0.2 g of dry plant material was extracted in 2 ml of distilled H_2_O after spiking with 25 μl of a 2 mg l^−1^ solution of d_6_-ABA as internal standard. After centrifugation (10,000*g* at 4 °C), supernatants were recovered, and the pH was adjusted to 3.0 with 30% acetic acid. The acidified water extract was partitioned twice against 3 ml of di-ethyl ether. The organic layer was recovered and evaporated under vacuum in a centrifuge concentrator (Speed Vac, Jouan, Saint Herblain Cedex, France). The dry residue was then re-suspended in a 9:1 H_2_O:MeOH solution by sonication. The resulting solution was filtered and directly injected into a UPLC system (Waters Acquity SDS, Waters Corp., Milford, MA, USA) interfaced to a TQD triple quadrupole (Micromass Ltd, Manchester, UK) mass spectrometer through an orthogonal Z-spray electrospray ion source. Separations were carried out on a Gravity C18 column (50 × 2.1 mm, 1.8 μm, Macherey–Nagel GmbH, Germany) using a linear gradient of MeOH and H2O supplemented with 0.1% acetic acid at a flow rate of 300 μl min^−1^. Transitions for ABA/d_6_-ABA (263 > 153/269 > 159) were monitored in negative ionization mode. Quantitation of plant hormones was achieved by external calibration with known amounts of pure standards using Masslynx v4.1 software.

### Soil water release curve construction

A soil moisture release curve was constructed by dew point psychrometry to estimate Soil water potential (ψ_soil_, MPa) from soil volumetric water content (θ_g_, g g^−1^). Six dry peat plugs were hydrated, and its volume and weight measured. The plugs were broke-down into a layer and placed in individual trays to facilitate homogeneous drying in a lab bench. At intervals determined by weight loss, soil in each cup was homogenised and a small volume (0.21 cm^3^) was placed in a sample holder cup and packed to match the average apparent density in the hydrated plugs. Soil and holder were weighed and inserted in a psychrometric chamber (C-52, Wescor, USA). Water potential was determined after at least 6 h of equilibrium. This was repeated until the samples were dried down to approximately soil volumetric water content θ_v_ = 0.10 g g^−1^ (Soil water potential ψ_soil_ = − 3 MPa). This θ_v_ corresponds to a plug weight of approximately 10 g. After each measurement, the sample and the holder were oven dried to constant weight to calculate the water content of each sample, which was used to determine θ_v_ at each point. The whole dataset was fitted to an exponential decay curve with Sigmaplot 12 (Systat Software Inc., San Jose, USA).

### Statistical treatment

Differences in parameters from the fitting functions were assessed by ANOVA (Table 1) with a model using Genotype as a fixed factor and the variable in X axis as a covariate. The significance of the interaction between the factor and the covariate is equivalent to the significance of the differences between slopes in the equations (ANCOVA).

## Additional files

**Additional file 1: Figure S1.** Scatter plot of Volumetric water content (A) and plug weight (B) versus Soil water potential and the parameters of the fitting function and the equation to transform plug weight to soil water potential (MPa). **Figure S2.** Variation in dry plug weight and saturated plug weight. **Figure S3.** Comparison of water loss from a saturated pot without shell or plant, with shell and with a shell and 4 weeks-old Col-0). Error bars denote standard deviation (N = 20). **Figure S4.** Diagram with shell measures and fitting with the plug. Photographs illustrating plants before an experiment.

**Additional file 2.** Photographs with the overlapping segmentation of the leaf area obtained as output of Easy Leaf Area to be processed by Leaf-GP.

**Additional file 3.** Excel with the whole output of Leaf-GP comprising parameters as individual leaf area, number of leaves and perimeters.

**Additional file 4.** Photographs with the overlapping segmentation of the leaf area obtained as output of Easy Leaf Area to be processed by Leaf-GP from a replicate experiment.

**Additional file 5.** Excel with raw data and calculation of the results of a replicate of the whole experiment.

## Data Availability

The datasets supporting the conclusions of this article are included within the article and its Additional files.

## Abbreviations

ABA: abscisic acid
DW: dry weight
LRWC: leaf relative water content
RGR: relative growth rate
RWC: relative water content
SDW: shoot dry weight
SFW: shoot fresh weight
SVWC: soil volumetric water content
SWP: soil water potential
TE: transpiration efficiency
TN: transpiration normalized
VPD: vapour pressure deficit
WT: wild type

## References

[1] Daszkowska-Golec A, Szarejko I (2013). Open or close the gate-Stomata action under the control of phytohormones in drought stress conditions. Front Plant Sci.

[2] de Ollas C, Dodd IC (2016). Physiological impacts of ABA–JA interactions under water-limitation. Plant Mol Biol.

[3] Qu X, Cao B, Kang J, Wang X, Han X, Jiang W (2019). Fine-tuning stomatal movement through small signaling peptides. Front Plant Sci.

[4] Vadez V, Kholova J, Medina S, Kakkera A, Anderberg H (2014). Transpiration efficiency: new insights into an old story. J Exp Bot.

[5] Hatfield JL, Dold C (2019). Water-use efficiency: advances and challenges in a changing climate. Front Plant Sci.

[6] Murata Y, Mori IC, Munemasa S (2014). Diverse stomatal signaling and the signal integration mechanism. Annu Rev Plant Biol.

[7] Bradford KJ (2008). Water relations and growth of the flacca tomato mutant in relation to abscisic acid. Plant Physiol.

[8] Rock CD, Zeevaart JA (1991). The *aba* mutant of *Arabidopsis thaliana* is impaired in epoxy-carotenoid biosynthesis. Proc Natl Acad Sci.

[9] Cutler SR, Rodriguez PL, Finkelstein RR, Abrams SR (2010). Abscisic acid: emergence of a core signaling network. Annu Rev Plant Biol.

[10] Easlon HM, Bloom AJ (2014). Easy leaf area: automated digital image analysis for rapid and accurate measurement of leaf area. Appl Plant Sci..

[11] Blum A, Tuberosa R (2018). Dehydration survival of crop plants and its measurement. J Exp Bot.

[12] De Ollas C, Arbona V, Gómez-Cadenas A, Dodd IC (2018). Attenuated accumulation of jasmonates modifies stomatal responses to water deficit. J Exp Bot.

[13] Blum A (2014). Genomics for drought resistance-getting down to earth. Funct Plant Biol.

[14] Puértolas J, Larsen EK, Davies WJ, Dodd IC (2017). Applying “drought” to potted plants by maintaining suboptimal soil moisture improves plant water relations. J Exp Bot.

[15] Granier C, Aguirrezabal L, Chenu K, Cookson SJ, Dauzat M, Hamard P, Thioux JJ, Bouchier-Combaud S, Lebaudy A, Muller B, Simonneau T, Tardieu F (2006). PHENOPSIS, an automated platform for reproducible phenotyping of plant responses to soil water deficit in *Arabidopsis thaliana* permitted the identification of an accession with low sensitivity to soil water deficit. New Phytol.

[16] Boyes DC, Zayed AM, Ascenzi R, McCaskill AJ, Hoffman NE, Davis KR (2001). Growth stage-based phenotypic analysis of Arabidopsis. Plant Cell.

[17] Zhou J, Applegate C, Alonso AD, Reynolds D, Orford S, Mackiewicz M (2017). Leaf-GP: an open and automated software application for measuring growth phenotypes for arabidopsis and wheat. Plant Methods.

[18] Jones HG (2007). Monitoring plant and soil water status: established and novel methods revisited and their relevance to studies of drought tolerance. J Exp Bot.

[19] Verslues PE, Agarwal M, Katiyar-Agarwal S, Zhu J, Zhu JK (2006). Methods and concepts in quantifying resistance to drought, salt and freezing, abiotic stresses that affect plant water status. Plant J.

[20] Saez A, Robert N, Maktabi MH, Schroeder JI, Rodriguez PL (2006). Enhancement of abscisic acid sensitivity and reduction of water consumption in arabidopsis by combined inactivation of the protein phosphatases type 2C ABI1 and HAB1. Plant Physiol.

[21] Caldeira CF, Bosio M, Parent B, Jeanguenin L, Chaumont F, Tardieu F (2014). Circadian rhythms of hydraulic conductance and growth are enhanced by drought and improve plant performance. Nat Commun.

[22] Sade N, Galkin E, Moshelion M (2015). Measuring arabidopsis, tomato and barley leaf relative water content (RWC). Bio-Protocol.

[23] Merilo E, Laanemets K, Hu H, Xue S, Jakobson L, Tulva I, Gonzalez-Guzman M, Rodriguez PL, Schroeder JI, Brosche M, Kollist H (2013). PYR/RCAR receptors contribute to ozone-, reduced air humidity-, darkness-, and CO2-induced stomatal regulation. Plant Physiol.

[24] Zandalinas SI, Balfagón D, Arbona V, Gómez-Cadenas A, Inupakutika MA, Mittler R (2016). ABA is required for the accumulation of APX1 and MBF1c during a combination of water deficit and heat stress. J Exp Bot.

[25] Coupel-Ledru A, Tyerman SD, Masclef D, Lebon E, Christophe A, Edwards EJ (2017). Abscisic acid down-regulates hydraulic conductance of grapevine leaves in isohydric genotypes only. Plant Physiol.

[26] Saez A, Apostolova N, Gonzalez-Guzman M, Gonzalez-Garcia MP, Nicolas C, Lorenzo O (2004). Gain-of-function and loss-of-function phenotypes of the protein phosphatase 2C HAB1 reveal its role as a negative regulator of abscisic acid signalling. Plant J.

[27] Koorneef M, Elgersma A, Hanhart CJ, van Loenen-Martinet EP, van Rijn L, Zeevaart JAD (1985). A gibberellin insensitive mutant of *Arabidopsis thaliana*. Physiol Plant.

[28] Durgbanshi A, Arbona V, Pozo O, Miersch O, Sancho JV, Gómez-Cadenas A (2005). Simultaneous determination of multiple phytohormones in plant extracts by liquid chromatography-electrospray tandem mass spectrometry. J Agric Food Chem.

