# Supplementary material for: A customizable method to characterize Arabidopsis thaliana transpiration under drought conditions
Source: Plant Methods. 2019 Aug 2;15:89. doi: 10.1186/s13007-019-0474-0 (PMC6676626; doi:10.1186/s13007-019-0474-0)
Supplement: Supplementary file 1 — Additional file 1: Figure S1. Scatter plot of Volumetric water content (A) and plug weight (B) versus Soil water potential and the parameters of the fitting function and the equation to transform plug weight to soil water potential (MPa). Figure S2. Variation in dry plug weight and saturated plug weight. Figure S3. Comparison of water loss from a saturated pot without shell or plant, with shell and with a shell and 4 weeks-old Col-0). Error bars denote standard deviation (N = 20). Figure S4. Diagram with shell measures and fitting with the plug. Photographs illustrating plants before an experiment. [file 13007_2019_474_MOESM1_ESM.docx]

Figure S1. Scatter plot of Volumetric water content (A) and plug weight (B) versus Soil water potential and the parameters of the fitting function and the equation to transform plug weight to soil water potential (MPa).

Figure S2. Variation in dry plug weight and saturated plug weight.

Figure S3. Comparison of water loss from a saturated pot without shell or plant, with shell and with a shell and 4 weeks-old Col-0). Error bars denote standard deviation (N=20).

Figure S4. Diagram with shell measures and fitting with the plug. Photographs illustrating plants before an experiment.
